# Impact of Huntington's across the entire disease spectrum: the phases and stages of disease from the patient perspective

**DOI:** 10.1111/j.1399-0004.2011.01748.x

**Published:** 2011-09

**Authors:** AK Ho, MB Hocaoglu, European Huntington's Disease Network Quality of Life Working Group

**Affiliations:** School of Psychology and Clinical Language Sciences, University of ReadingReading, UK

**Keywords:** emotional well-being, functional ability, Huntington's disease, physical, quality of life, social

## Abstract

Ho AK, Hocaoglu MB for the European Huntington's Disease Network Quality of Life Working Group. Impact of Huntington disease across the entire disease spectrum: the phases and stages of disease from the patient perspective.

Although Huntington's disease (HD) is a neurodegenerative disease characterized by motor, cognitive and behavioural disturbances, there has been little empirical data examining what patients are most concerned about throughout the different stages of disease, which can span many years. Semi-structured face-to-face interviews were individually conducted with 31 people living with different stages of Huntington's, from pre-clinical gene carriers to advanced stage. We examined how often participants raised issues and concerns regarding the impact of Huntington's on everyday life. The Physical/functional theme hardly featured pre-clinically, but was strongly present from Stage 1, rose steadily and peaked at Stage 5. There were no significant changes between stages for the Emotional, Social, and Self themes that all featured across all stages, indicating that these issues were not raised more frequently over the course of the disease. Likewise, the more rarely mentioned Financial and Legal themes also remained similar across stages. However, the Cognitive theme only featured between Stages 1 and 4, and hardly at all pre-clinically and at Stage 5. These findings provide insight into patients' important and unique perspective and have implications for the management and development of interventions across the spectrum of HD stages.

Section Editor: Aad Tibben, email: a.Tibben@lumc.nl

Huntington's disease (HD) is a hereditary autosomal dominant neurodegenerative disease for which direct mutation predictive testing has been available since 1993. Although the motor, cognitive and behavioural disturbances associated with HD have been well documented in the literature, there is little empirical data examining how this translates into what patients themselves are concerned about throughout the long course of disease. The profound impact on patients' physical and also psychosocial well-being (1, 2) has been showed through data from generic quality of life questionnaires. While these generic measures are useful, they also arbitrarily constrain the information provided, and hence limit our knowledge of patients' experience of living with this complex disease. Interview studies which allow patients to freely describe the impact of disease play an important role in providing a meaningful understanding of patients' perspective on their own well-being. The accumulating body of qualitative interview research in HD has focussed on specific topics or specific patient subgroups (3, 4). Information on the everyday impact of HD and patient experiences at different stages of HD is scarce. This study seeks to investigate how HD affects the experience of everyday life in order to understand what types of issues are at the forefront of patients' minds, and how the profile of concerns may evolve throughout the full trajectory of illness from pre-clinical to end-stage HD.

## Methods

### Participants

Eighty individuals from pre-clinical status (Pre-HD) to late-stage HD were invited to participate; 31 people volunteered within the time frame of the study and provided consented to participate. Pre-HD individuals were HD mutation carriers identified by the predictive test who reported that they have not yet been given a diagnosis of manifest HD. The entire clinical disease spectrum was represented, and operationalized according to self-reported Shoulson and Fahn (5) severity staging, ranging from Stage 1 (i.e. able to function at home and work) to Stage 5 (i.e. require full-time caring and nursing). All participants were from the UK and comprised Pre-HD (3), Stage 1 (5), Stage 2 (5), Stage 3 (3), Stage 4 (9) and Stage 5 (6) individuals; 67.7% were females, 32.3% males, 61.3% were aged between 30 and 59 years, and 38.7% between 60 and 89 years.

### Data collection and analysis

Semi-structured interviews were conducted at participants' homes and lasted approximately 60 min. Interview questions were standardized using open-ended questions, followed by probes. These probes addressed areas indicated by the health-related quality of life concept as defined by emotional well-being, spirituality, sexuality, social functioning, family life, occupational functioning, communication, eating, functional ability, physical status, treatment satisfaction, self-esteem, body image, future orientation, global ratings of health and life satisfaction (6). Pictorial topic cards were also used to facilitate the interview process and support communication with Stage 5 participants since this has been shown to be useful in previous research (7). Interviews were recorded, transcribed verbatim, and classified according to themes that emerged, i.e. (i) Physical/functional, (ii) Cognition, (iii) Emotion, (iv) Social, (v) Self, (vi) Legal and (vii) Financial. Detailed qualitative analysis of interview narratives was beyond the scope of this brief report.

**Figure 1:**
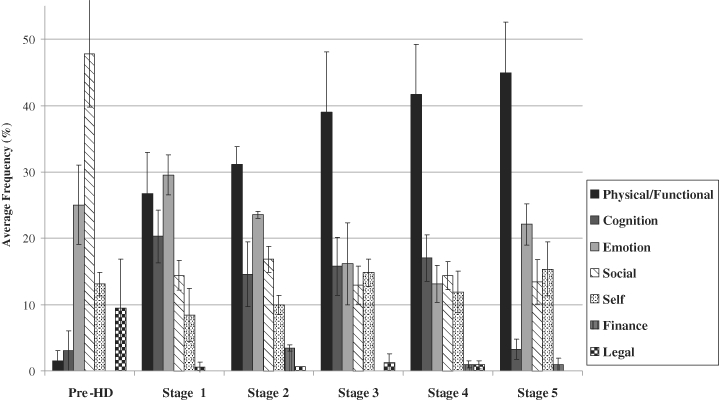
Average frequency (%) for each theme according to Huntington's disease stage.

In this study, the number of times each participant mentioned specific instances where HD had a negative impact on their lives was classified according to the seven themes. For each participant, the frequency with which they raised issues relating to each theme in interview was summed according to theme and then divided by the total number of reports across all themes to be expressed as a percentage frequency for each specific theme. In order for meaningful comparisons to be made across different disease stages, the percentage frequency of reporting each theme was then averaged across the number of participants in each stage of disease. Therefore, Fig. 1 shows the average percentage frequency of participants' raising of each theme for each disease stage.

## Results and discussion

As shown in Fig. 1, most of the issues raised by pre-HD participants fell into the Social theme (i.e. family relationships in the context of HD, dissatisfaction with Huntington's services, lack of support (family, friends, health professionals, and community), and others' perceived attitude to HD and lack of awareness regarding Huntington's), followed by the Emotional theme (i.e. anxiety regarding the impact of Huntington's on the family and also themselves in terms of emerging HD signs and symptoms). Some of these latter concerns resonated with the Self theme (i.e. acceptance of HD, self-confidence, independence, and especially fulfilling personal wishes). Legal issues were raised less frequently than other themes, but seemed to feature strongly in pre-HD relative to other stages, as the implications of the disease led to making appropriate arrangements up front, particularly regarding insurance. Physical/functional and Cognitive issues hardly featured at all in pre-HD.

In Stage 1, Physical activities and functional ability (i.e. difficulty in sleeping well, swallowing food and drink, and experiencing twitches and jerks), Cognitive issues (e.g. difficulty in concentrating, organizing, deciding, slower in getting things done, and particularly difficulty in remembering everyday information), Emotional issues (low mood, low motivation, and particularly anxiety about the emergence of signs and symptoms associated with HD) were frequently reported. Social (complicated family relationships in the context of HD, lack of support and awareness of HD from those around them, as well as concerns regarding going out to socialize) and Self (fulfilling personal wishes and changes in self-confidence) themes also appeared.

For Stage 2 participants, Physical/functional (i.e. swallowing food and drink, sleeping well, driving performance, maintaining a stable body weight, changes with balance, walking, clarity of speech pronunciation, and writing ability), Emotional (i.e. low mood, low motivation, anxiety about signs and symptoms associated with HD, losing patience, mood swings, and worries regarding the impact of Huntington's on the family), Cognition (i.e. difficulty in remembering everyday information, organizing daily activities, changes in spoken language expression, ability to learn new things and multitasking), Social (complicated family relationships in the context of HD, going out for social occasions, maintaining sexual relationships, others' attitude to HD and lack of awareness regarding Huntington's, and dissatisfaction with Huntington's services) and Self (i.e. fulfilling personal wishes, changes in self-confidence, embarrassment and being self-conscious) themes featured. The Financial theme (mainly issues surrounding change or loss of employment) occurred less frequently, and occurred even less in subsequent stages.

In Stages 3 and 4, the Physical/functional theme (i.e. sleeping well, swallowing, tiredness, speech pronunciation, walking, stair climbing and handling objects, driving, balance, chorea, slowness, dressing, washing and slowness, and also incontinence in the latter stage) was more dominant than other themes of Cognition (i.e. concentrating, remembering everyday information, slow to get things done, making decisions, spoken language expression, learning new things and time orientation), Emotion (i.e. anxiety over HD signs and symptoms, low motivation, for the latter stage low mood and mood swings, and anxiety regarding the impact of Huntington's on the family), Social (key issues at Stage 3 were regarding going out for social occasions, others' attitude to HD and lack of awareness regarding Huntington's, and maintaining sexual relationships; and at Stage 4, ineffective communication, others' attitude to HD and lack of awareness regarding Huntington's, family relationships in the context of HD, lack of support from family, friends, and health professionals, going out to socialize occasions and maintaining sexual relationships), and Self (i.e. reduced confidence, independence, fulfilling personal wishes, feeling embarrassed or self-conscious, and for the latter stage the acceptance of HD in one's life).

The dominance of the Physical/functional theme (i.e. key issues were ambulation, and swallowing; also raised was feeling tired and sleeping well, issues regarding maintaining body weight, clarity of speech, movement control, and writing and dressing) was clear in Stage 5, while the Cognitive theme hardly featured. Low mood was the most frequently raised Emotional aspect in Stage 5, with only a few individuals mentioning low motivation, feeling angry and worrying about HD symptoms. The key Social issue for most Stage 5 participants was the lack of support from family, friends and health professionals. Like other stages, the Self theme was represented mainly by issues with self-confidence, self-consciousness, and personal wishes.

Using Kruskal–Wallis nonparametric one-way anova, the effect of stage was examined for each theme. The occurrence of Emotion, Social, Self, Finance and Legal themes were not significantly different between groups, however the Physical/functional (*H*(5) = 12.70, p < 0.05) and cognitive (*H*(5) = 11.40, p < 0.05) themes showed significant differences. *Post hoc* analyses showed that for the Physical/functional theme, there was a significant difference between pre-HD and Stage 1 (*U* = 0.00, p < 0.05), but not for consecutive groups at subsequent stages, and only a trend between Stage 1 and Stage 5 (*U* = 5.00, p < 0.07). This indicates that there was a significant increase in Physical/functional issues at Stage 1, and this then remains equally high after this, with no real significant increase between each subsequent severity stage. For the Cognitive theme, there was a mild trend for an increase between the pre-HD and Stage 1 group (*U* = 2.00, p < 0.09), with no significant differences between subsequent stages, until Stage 4 which was significantly greater than Stage 5 (*U* = 7.50, p < 0.05). Thus, there was a flat inverted *U* curve with Cognitive issues appearing from Stage1 and remaining at a similar low level of occurrence until Stage 5, where they dissipated.

## Conclusion

Our frequency of patient mention data provide unique insight into the impact and concerns of a wide range of patients, and the different trajectories of development across the different themes over the course of HD. There appeared to be four phases of HD marked by different profiles of HD impact, which synchronize with and correspond coherently with key HD-related milestones. During the pre-HD stage, participants were pre-occupied with internal and relational issues; these Emotional, Social and Self concerns remained throughout the subsequent stages of HD and did not increase. In the early stage of manifest HD (Stages 1 and 2), often marked by clinical diagnosis and practical consequences of disease, participants' comments frequently revolve around Physical/functional and Cognitive issues, in recognition and perhaps also adaptation to the emergence of concrete HD symptoms. During moderate HD (Stages 3 and 4), there was least change in the state of play for the various thematic profiles; therefore from the patients' perspective, this appeared to be a period of stability in the overall scheme of disease progression. Because of the very gradual, but inexorable increase in Physical/functional concerns, late-stage HD (Stage 5), was characterized predominantly by this theme, and the lack of Cognitive concerns most likely due to degree of cognitive impairment which affects insight (8), while internal and relational (Emotional, Social and Self) themes persisted without much change throughout the preceding severity stages. In Parkinson's disease another protracted neurodegenerative disease, physical and social aspects of health-related quality of life, although not cognitive, were found to deteriorate over time in a 4-year longitudinal study (9). The physical and functional aspect was also dominant in Alzheimer's disease (10).

One of this study's limitations is the cross-sectional design, which nevertheless served to provide for the first time a snap-shot of concerns in a wide spectrum of patients. Furthermore, qualitative scrutiny of participants' detailed comments, outside of the scope of this report, will certainly supplement the perspective gained by the current analysis. It may also be relevant to consider the data in the context of age since more severely affected patients tend to be older than presymptomatic and mild patients due to the protracted course of the disease. Our sample also comprised more women than men, and it is not clear if gender might differentially affect health-related quality of life in HD. In Parkinson's disease, it is worth noting, however, that men and women are no different (11).

In summary, this study provides for the first time, an overview of the profile of HD impact throughout the full trajectory of illness from pre-clinical to end-stage HD, from patients' unique and important perspective. Information regarding phases of change and stability over the protracted course of the disease will be of interest to clinicians, researchers and patients. It also provides an informed basis for the long-term management of health and well-being in HD, and the development of interventions across the spectrum of HD stages.

## Appendix

European Huntington's Disease Network Quality of Life Working Group: Walter Bucher, Alyson Bradbury, Beatrice de Schepper, John Eden/Sue Beevers and team, George El-Nimr, Victor Hendrikx, Alis Hughes, Diana King, Ursula Kleibrink, Rita Kuttruff-Wilschut, Bernhard Land- wehermeyer, Anne Lenon-Bird/Catherine Paradise and team, Christiane Lohkamp, Rhona MacLeod, Helene Padieu, Marie-Odile Perrousseaux, Asuncion Martinez, Lilliane Rapaille, Helen Santini, Pavla Sasinkova, Beverley Soltysiak, Steve Smith, Hans van der Leer, Lucienne van der Meer, Jo Anne Watton and team, Marleen van Walsem, Michael Wooldridge, Paola Zinzi.
